# Understanding posttraumatic growth in palliative care: a qualitatively driven mixed-methods study using patients’ life narratives

**DOI:** 10.1186/s12904-026-02147-4

**Published:** 2026-05-26

**Authors:** Laura Galbiati, Elsa Baader, Emilie Bovet, Gian Domenico Borasio, Emmanuelle Poncin, Jean-Baptiste Mercoli, Fatoumata Diawara, Agnieszka Grosjean-Bagnoud, Mathieu Bernard

**Affiliations:** 1 Palliative and Supportive Care Service, Lausanne University Hospital and University of Lausanne, Lausanne, Switzerland; 2 HESAV - School of Health Sciences – Vaud and HES-SO University of Applied Sciences and Arts Western Switzerland, Lausanne, Switzerland; 3 Palliative Care unit, Valais Hospital, Martigny, Switzerland; 4 La Maison Azur, Palliative Care Home, Sion, Switzerland; 5 Palliative Care Unit, Oncology Department, Gruppo Ospedaliero Moncucco, Lugano, Switzerland

**Keywords:** Posttraumatic growth, Quality of life, Palliative care, Psychological changes, Mixed-methods, Narrative method

## Abstract

**Background:**

Numerous studies have revealed that the shock engendered by a serious illness can lead to positive psychological changes known as posttraumatic growth (PTG). Most studies examined this phenomenon in early-stage cancer and with the Posttraumatic Growth Inventory (PTGI). Our current understanding of the psychological processes behind PTG remains limited. Through life narrative interviews, our study aims (i) to describe profiles of psychological changes of palliative care patients and to highlight characteristics of these changes, and (ii) to link these profiles with quality of life and PTGI.

**Methods:**

This study adopted a multi method design. Semi-structured interviews were conducted with patients based on the Biographic Narrative Interpretive Method (BNIM) in three centres in Switzerland. An ideal-type analysis was used to establish ideal-types of changes and a thematic analysis to highlight characteristics of these changes. In addition, posttraumatic growth and quality of life were assessed through the PTGI and the McGill Quality of Life-Revised questionnaire (MQOL-R).

**Results:**

In total, 23 patients participated (13 female, median age = 73). The ideal-type analysis identified four types of psychological changes: (i) “Overcoming adversity” (*n* = 6), (ii) “Resilience” (*n* = 7), (iii) “Resigned acceptance” (*n* = 3), (iv) “Psychological suffering” (*n* = 7). “Overcoming adversity” and “Resilience” showed higher PTGI scores than “Resigned acceptance” and “Psychological suffering”. “Overcoming adversity”, “Resilience” and “Resigned acceptance” showed higher MQOL-R scores than “Psychological suffering”.

**Conclusions:**

Regarding research, life stories offer an innovative way to understand and highlight patient’s psychological profiles shaped by past experiences and surpassing the limits of questionnaires. In term of clinical perspective, narrative techniques, known to promote meaning-making, offer valuable opportunities to consider both past and present experiences of patients and thus understand and support their psychological adaptation to face serious illness at the end-of-life.

## Background

Palliative care adopts a holistic approach, addressing somatic, psychological, social, and spiritual dimensions [1]. While medical advances alleviate somatic suffering, patients and families in palliative care often face significant psychological distress, particularly at the end of life [2]. This distress arises from grief, loss of autonomy, shifting social identity, and confronting mortality. Meta-analyses report that 32–39% of cancer patients experience psychiatric disorders [2–4]. Research highlights that trauma, such as a cancer diagnosis, can trigger meaning-making processes through which individuals attempt to integrate and make sense of the illness experience [5] These processes may lead to a range of positive psychological outcomes, including posttraumatic growth (PTG), benefit-finding, a strengthened sense of coherence, enhanced appreciation of life, and improved relational quality [2, 4]. PTG reflects a transformative process born from inner struggles after trauma [6, 7]. Engaging in PTG may be emotionally distressing and challenging, even if the result is positive [7].

Most quantitative studies on posttraumatic growth (PTG) have utilized the 21-item PTGI [8], which assesses changes in personal strength, relationships, life possibilities, life appreciation, and spirituality. The PTGI relies on retrospective perceptions of positive change in predefined areas and may be tainted by a positivity bias linked to social desirability [9]. More broadly, recent literature suggests that self-reported PTG may partly reflect “illusory” growth, that is, perceived changes shaped by cognitive biases, defensive coping processes, and culturally shared expectations about the positive meaning of adversity, rather than genuine psychological transformation [10]. Such processes may contribute to an overestimation of PTG when relying solely on self-report measures, highlighting the importance of complementary approaches to capture the complexity of psychological change.

Reviews of qualitative studies have highlighted additional domains that remain insufficiently represented in the PTGI, such as changes in the relationship to the body and physical functioning, the experience of symptoms and treatment, as well as identity reconfiguration in the face of illness [11]. More recent work has further emphasized dimensions of “physical posttraumatic growth”, including increased bodily awareness, redefined limits, and new relationships to health and vulnerability [12]. These findings underline the importance of approaches capable of capturing experiential dimensions that extend beyond predefined questionnaire categories.

Research has largely focused on early-stage cancer patients, breast cancer patients, and survivors [11, 13, 14], leaving palliative care experiences underexplored. Moreover, most oncology and palliative care studies have relied on cross-sectional or retrospective quantitative designs, which present important drawbacks for investigating a psychological phenomenon of transformation. These studies offer limited insight into the mechanisms of PTG. Life narratives provide a promising method to explore PTG processes and dynamics. Recently applied in oncology, they have helped identify specific linguistic profiles of cognitive and emotional processes in PTG narratives [15].

Using life narrative interviews, the primary aim of this study is to highlight the nature of the psychological changes experienced, including posttraumatic growth, in the context of serious illness and the end of life by drawing on the patients’ life stories and exploring the changes experienced since the onset of the illness. The secondary aim is to compare these qualitative findings with quantitative assessments of quality of life and posttraumatic growth, measured through standardized questionnaires and analyzed in relation to ideal-types.

## Methods

This is a mixed-methods exploratory study, deploying qualitative methods to test our primary aim, and quantitative methods to test our secondary aim. The combined use of qualitative and quantitative methods aimed to explore the concordance or potential discrepancies between the findings from these two approaches.

### Recruitment, screening and informed consent procedure

The project was approved by the ethical committee of the canton de Vaud in August 2021 (2021 − 01522). The data collection was conducted in the Palliative and Supportive care service of the Lausanne University Hospital (palliative care unit and mobile palliative care team), the Hospital of Valais, (palliative care unit and mobile palliative care team), and the inpatient hospice “Maison Azure”. A referral physician identified eligible patients according to the following criteria:

- Inclusion criteria: age ≥ 18; cancer diagnosis or incurable illness with reduced life expectancy; enrolled in palliative care; clinical state enabling the person to take part in a one to two-hour interview.
- Exclusion criteria: cognitive or psychiatric disorders preventing individuals from giving informed consent; communication problems (e.g. foreign language not spoken by members of the research team, deafness).

After identification by the palliative care team, patients were approached by an independent research collaborator who informed them about the study objectives and obtained written consent. Two meetings were scheduled with each patient: the initial meeting was dedicated to collecting socio-demographic data and the life narrative (see supplementary material), followed one week later by a second meeting assessing posttraumatic growth and quality of life using validated measures. On average, each interview last between 1 and 2 h. In addition to the posttraumatic growth and quality of life questionnaires, two further instruments were administered in the second meeting to assess the overall impact of the life story intervention on participants’ quality of life and stress levels. The inclusion of these additional questionnaire was primarily motivated by ethical considerations. Given the sensitive context of end-of-life care, we sought to ensure that participation in a life narrative interview did not generate undue psychological distress. These measures were intended to monitor participants’ subjective experience and to assess the perceived impact of the procedure. The questionnaire included brief self-report items using a 5-point Likert scale for perceived impact on quality of life and a 0–10 scale to assess subjective stress following the life story interview. These data were not included in the main analyses, as they were collected primarily for ethical monitoring and safety purposes rather than as study outcomes.

The interviews were audio recorded and transcribed verbatim. Patients were free to withdraw at any time, without giving a reason.

### Life narrative interview and quantitative measures

The Biographic Narrative Interpretive Method (BNIM) is a qualitative research method aiming to analyze three interconnected dimensions of human experience: a person’s life story (biography), the way it is told (narrative) and its social interpretation (interpretive) [16]. This method considers multiple factors that can influence the narrative, its interpretation and transmission, while paying particular attention to the historical context and associated subjectivity. BNIM facilitates the emergence of narratives through three steps: (i) the researcher asks an open-ended question about the participant’s life story. We used the following open-ended question: *“ As part of this project on life narratives*,* I’d like you to share your life story*,* especially regarding health and illness. Take your time; I’ll listen without interrupting and may ask questions afterwards”*; (ii) using the participant’s exact words, the researcher asks them to elaborate on specific scenes or experiences from their initial narration; (iii) the researcher asks targeted questions about changes since the onset of the illness.

### Quantitative measures

Sociodemographic data included age, sex, mother tongue, education, marital status, profession, religion and spirituality. Medical data comprised primary diagnosis and comorbidities.

The Posttraumatic Growth Inventory (PTGI) [8] aims to evaluate posttraumatic growth through a 21-item scale including a total score (0–105) and five subscales: better relationship with others (0–35), new possibilities (0–25), personal strengths (0–20), spiritual changes (0–10), and appreciation of life (0–15) with higher scores indicating higher post-traumatic growth. We used a translated French version [17].

Quality of life was assessed by the French version of the McGill Quality of Life-Revised questionnaire (MQOL-R) [18], which was specifically developed for use with people suffering from terminal illness [19]. It contains 14 items (from 0 to 10, a higher score reflecting better quality of life), giving an overall total score, and quality of life scores for the following four subscales: physical, feelings and thoughts, existence and relationship (range from 0 to 10). The tool starts with a single item assessing overall subjective QoL (range 0–10).

### Analyses

#### Qualitative analyses

To address our main research question, two complementary qualitative analytic approaches were conducted: an ideal-type analysis and a codebook-based thematic analysis. Ideal-type analysis is a qualitative method used in the social sciences to simplify complex realities by constructing typologies that highlight distinct patterns of behaviors, thoughts, and emotions, with the aim of representing the essential characteristics of a social phenomenon.

In addition, a codebook-based thematic analysis was conducted for each ideal-type to identify recurrent patterns of psychological and existential changes across participants. This approach allowed us to explore similarities and differences in content across the ideal-types and to provide a more detailed characterization of the themes associated with each profile identified through the ideal-type analysis.

##### Ideal-type analysis

Ideal-type analysis was used to identify ideal-types related to the changes experienced before and during the illness. The research team followed the data analysis process described by Stapley et al. [20]. The coding procedure is divided into 7 stages: (i) becoming familiar with the dataset by listening to the audio recorded interview and reading the transcripts (E.Ba, E.Bo, L.G, M.B); (ii) writing the case reconstructions by summarizing each interview transcript in its entirety (E.Ba); (iii) constructing the ideal-types by identifying patterns across the dataset (E.Ba); (iv) identifying the optimal cases by identifying the case best illustrating the characteristics of each group (E.Ba, E.Bo, L.G, M.B); (v) forming the ideal-type descriptions, by considering the main characteristics and then name the group (E.Ba, E.Bo, L.G, M.B); (vi) checking credibility by sending the work to an independent researcher for cross-checking in order to ensure that the descriptions of each group are meaningful and differentiated enough (E.Bo); (vii) comparing ideal-types between each group and highlighting the differences and similarities of cases within each group (E.Ba, E.Bo, L.G, M.B).

The ideal-types should be understood as heuristic analytic constructs rather than empirically existing or normative patient profiles. Their constructions was guided by several interpretative dimensions, including the presence of posttraumatic growth-related changes, the mobilisation of resources, the level of psychological or existential distress, and the participants’ relationship to illness (shock, adaptation, resignation), as well as the narrative tone and structure of their accounts.

Illustrative cases were selected based on their proximity to the core characteristics of each ideal-type and were identified through iterative team discussions to enhance conceptual clarity. Importantly, some cases presented features overlapping multiple ideal-types. These borderline cases were collectively discussed and contributed to refining the typology, suggesting that the ideal-types should be interpreted as points along a continuum rather than mutually exclusive categories.

##### Thematic analysis

Thematic analysis was conducted using a codebook-based approach (often referred to as applied thematic analysis; (Guest, 2012) [21], aiming to systematically identify patterns across participants’ narratives. While thematic analysis was originally outlined by Braun and Clarke [22], the present study follows a structured, codebook-oriented approach rather than a reflexive thematic analysis as developed in their later work (Braun & Clarke) [23, 24].

Initially, three researchers (L.G, E.Ba, E.Bo) independently coded five interviews without a pre-established coding framework, allowing themes and categories to emerge inductively from the data. This inductive approach was chosen to remain as close as possible to participants’ accounts and to avoid imposing predefined categories.

Following this first phase, the researchers compared their respective coding and developed a shared codebook, synthesizing and harmonizing the categories identified individually. This codebook served as a structuring guide for the remainder of the analysis and was iteratively refined as new interviews were coded, allowing the integration of new themes and the adjustment of existing categories.

To enhance consistency in code application, the interviews were subsequently coded by two researchers (L.G, E.Ba), and discrepancies were discussed and resolved. In addition, a third researcher (E.Bo) conducted systematic cross-checking of the coded material to identify inconsistencies, omissions, or divergences in interpretation.

This analytic approach, combining inductive coding with iterative codebook development and cross-checking procedures, is consistent with a codebook/reliability-oriented form of thematic analysis. It focuses on identifying recurrent patterns across the dataset rather than on a fully reflexive and interpretative analysis of individual meaning-making.

To further identify the most salient themes within each ideal-type, we calculated their frequency. Themes were retained when they were mentioned by at least half of the participants within each ideal-type.

#### Quantitative analysis

We used descriptive analyses (median and interquartile ranges) for the PTGI and for the McGill Quality of Life Revised questionnaire scores by considering the four ideal-types. Descriptive trends are preliminary; statistical testing was precluded by sample size constraints.

### Positionality of the researchers

Data were collected by two psychologists and a researcher in political science, and analyzed by a multidisciplinary team including psychologists and an anthropologist, all with training in qualitative methods and experience in palliative care research. This background may have facilitated a sensitive exploration of participants’ narratives, particularly regarding psychological and existential dimensions such as meaning-making and posttraumatic growth. At the same time, the team was aware that its disciplinary perspectives, primarily rooted in the human and social sciences, may have shaped both data collection and interpretation, potentially placing greater emphasis on subjective and narrative aspects, while other dimensions, such as clinical or symptom-related experiences, may have been less foregrounded.

## Results

### Recruitment

Initially, 28 participants were eligible to take part in the study. All gave their consent. Five participants withdrew during the study for the following reasons: incomprehension of the study and loss of meaning (*n* = 1), inability to speak French (*n* = 1), unstable symptoms (*n* = 1), cognitive troubles (*n* = 1), unknown reason (*n* = 1). 23 patients participated in the entire study: nine patients were recruited by the mobile team of the Lausanne University Hospital, eleven patients in the hospital of Valais, and three patients in the inpatient hospice “Maison Azure” in Sion.

### Descriptive data of the sample

The socio-demographic and medical data are shown in Table 1.

**Table 1:** Patients’ socio-demographic and medical data (*n* = 23)

Variables of the entire sample (*n* = 23)		
Age		
Median	70	
Inter quartile range	66–83	
	*n*	***%***
Sex		
Female	13	57
Male	10	43
Mother tongue		
French	21	91
German	1	4
Italian	1	5
Marital status		
Single	1	4
Married	10	44
Divorced or separated	6	26
Widow	5	22
Missing data	1	4
Education level		
Primary/secondary school	4	18
Vocational school/Apprenticeship	8	35
High school	2	9
Technical school	1	4
University/Higher education	6	26
Other	1	4
Missing data	1	4
Primary diagnosis		
Cancer	13	57
Heart disease	3	13
Amyotrophic lateral sclerosis	2	9
Other	4	17
Missing data	1	4

### Ideal-types of changes and illustration of themes and sub-themes

Using ideal-type analysis, the research team identified four ideal types. For each type, we highlighted themes related to psychological and existential changes. Some themes captured broader, more global transformations, while more specific sub-themes emerged from these overarching patterns. To maintain conciseness, each sub-theme is illustrated with a single representative quotation.

#### Ideal-type 1: “overcoming adversity” (*n* = 6 patients)

**Fig. 1:**
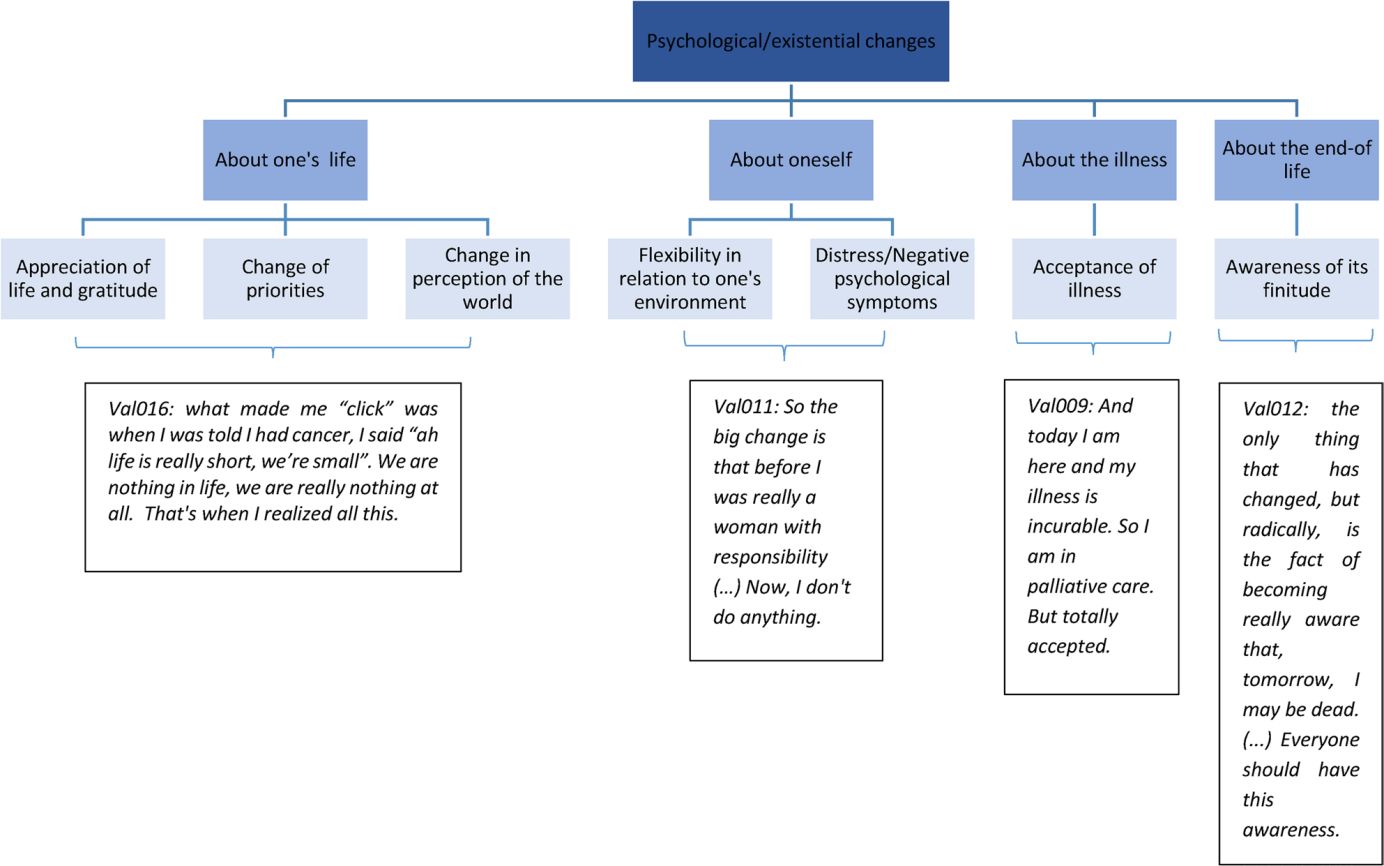
Codebook-based thematic analysis of ideal-type 1: “overcoming adversity”

This ideal-type’s narrative is generally chronological. Their tone is rather soothing. Most participants talk about their lives in general terms, without going directly into the illness. This ideal-type groups participants who mention observable and concrete positive changes after the illness in at least one of the five dimensions of posttraumatic growth (relationships with others, new possibilities, personal strength, spiritual change and appreciation of life). Despite difficult experiences in their lives before or during the illness (trauma, death, complicated treatments, side effects, pain, etc.), these patients have many resources for coping with the illness. Some of them have projects that pull them forward, they are able to hold on to the little things in life. All perceive strong support from their relatives, friends, and medical professionals. Most of these patients report feeling no shock when the disease was announced. Others took time to realize it. But in all cases, the disease changed their vision of the world or of their priorities. It changed their values and brought them closer to their loved ones. All emphasize the quality of the care they received, not necessarily throughout their journey, but at least in palliative care. They are calm and stress the positive aspects of their illness. All of them regularly mention the people around them and the people who have meant a great deal to them in their lives and during their illness Figure 1 illustrates the themes related to psychological and existential changes of ideal-type 1 “Overcoming adversity”.

#### Ideal-type 2: “resilience” (*n* = 7 patients)

**Fig. 2:**
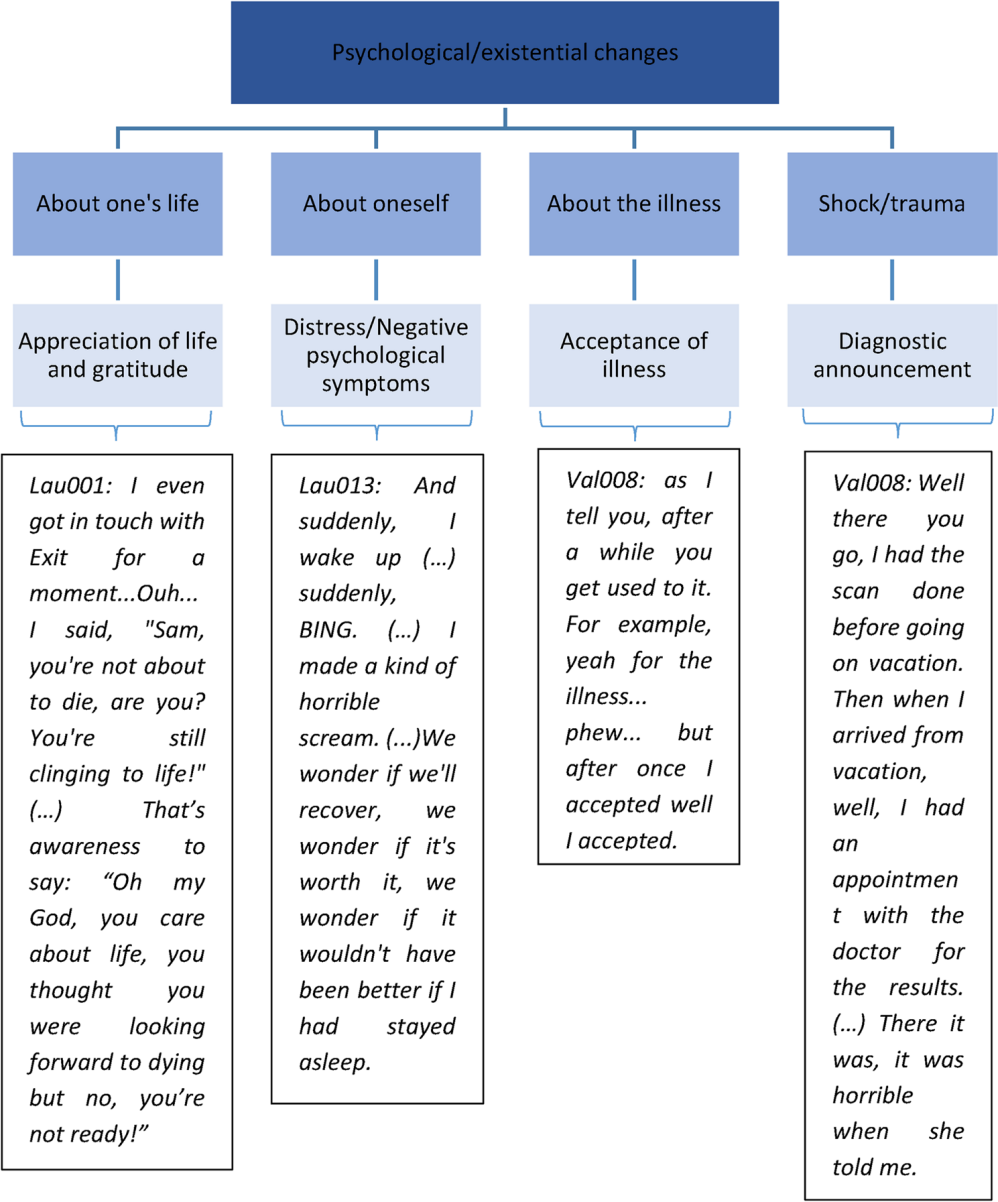
Codebook-based thematic analysis of ideal-type 2: “resilience”

The way people classified in this ideal-type tell their story varies: some quickly describe the symptoms and illness, while others take a long time to get to the illness episode. This ideal-type groups together patients who have not observed any changes related to the illness in the sense of posttraumatic growth, but who nevertheless have several resources within them. Patients of this ideal-type do not necessarily report shock or trauma, or a particularly difficult path to recovery. All these patients can count on the support of their families, but also on their optimism and desire to move forward. Most of them experienced shock when the disease was announced, as well as feelings such as revolt, injustice or surprise. Still, they focus on the positive, seem to keep their spirits up, and have daily routine. Figure 2 illustrates the themes related to psychological and existential changes of ideal-type 2 “Resilience”.

#### Ideal-type 3: “resigned acceptance” (*n* = 3 patients)

**Fig. 3:**
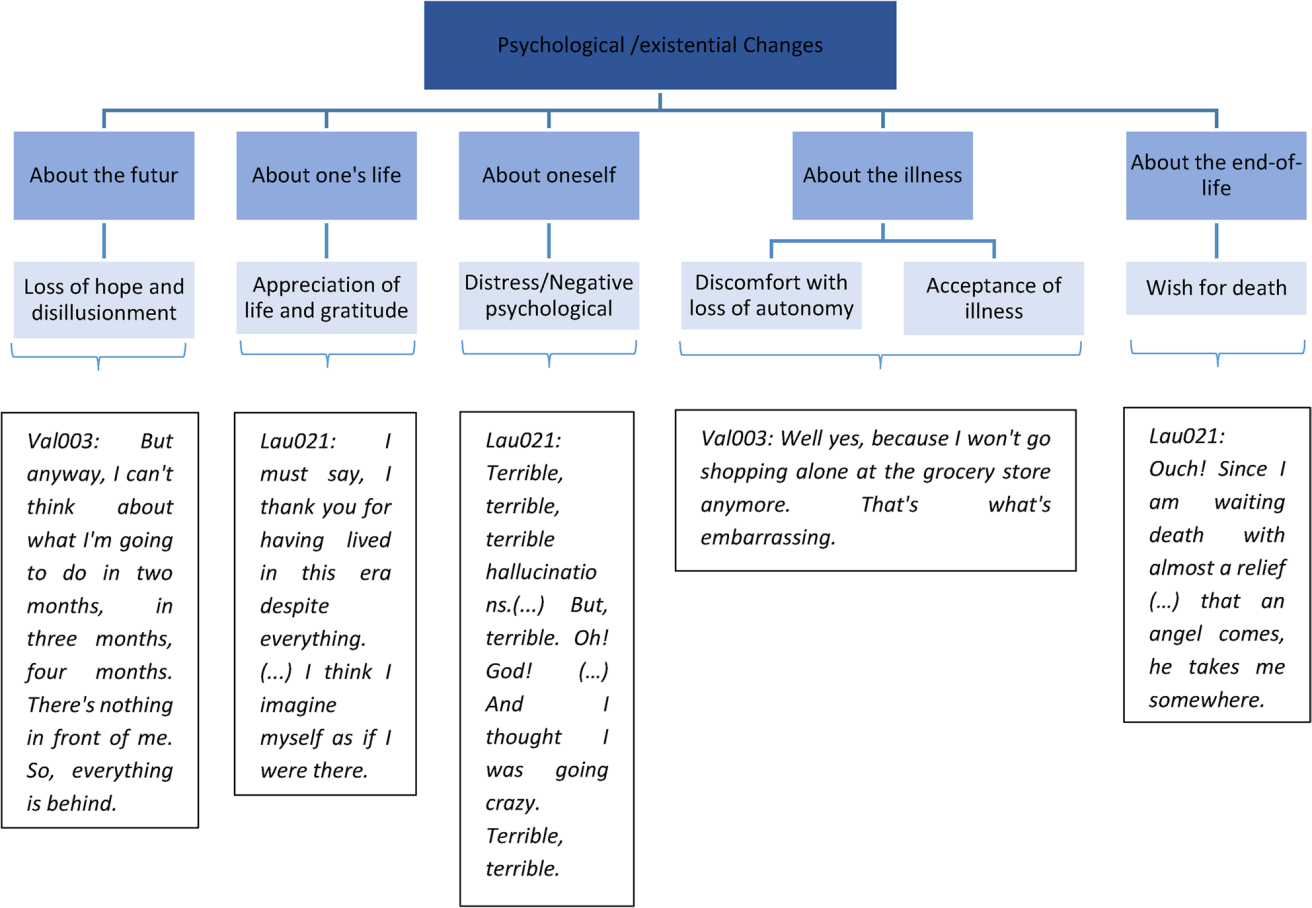
Codebook-based thematic analysis of ideal-type 3: “resigned acceptance”

This ideal-type’ narrative quickly addresses issues related to the illness, and their stories are rather brief. The patients grouped in this ideal-type express that they no longer wish to live, although their reasons are different. For some, passing away would enable them to avoid being a burden for their loved ones. Others feel that it’s simply time for them to go. They appear ready and serene. Participants also experienced a radical change in their perception of palliative care. At first, they refused such care, which they understood as being synonymous with death. They then realized how much palliative care could bring them in terms of comfort. All patients emphasize their satisfaction with the medical support they receive and identify their families are their main resource. They report no positive changes as a result of their illness. Rather than reflecting a positive or integrative acceptance, this profile appears closer to a form of resigned acceptance, characterized by a withdrawal from future-oriented perspectives and, in some cases, underlying psychological distress. Figure 3 illustrates the themes related to psychological and existential changes of ideal-type 3 “resigned acceptance”.

#### Ideal-type 4: “ psychological suffering ” (*n* = 7 patients)

**Fig. 4:**
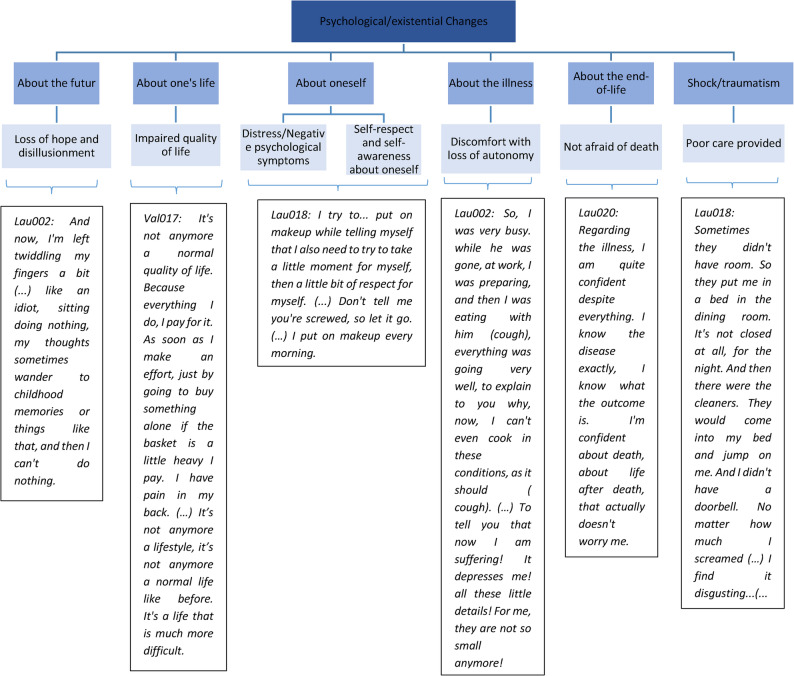
Codebook-based thematic analysis of ideal-type 4: “psychological suffering”

The narrative of this ideal-type is chronological. The stories told by patients in this group highlight clearly identified psychological and/or existential suffering. Levels of distress vary between patients. Their narratives are marked by grievances, suffering, and loss (of interest, desire, motivation, pleasure, etc.). Some patients’ lives can be summed up as a succession of traumas or negative events at each stage of their lives. Most of them have lost faith in their god after experiencing all these different trials of life. Symptoms of depression and anxiety also emerge in some stories. The only resource they emphasize is having children. They highlight the poor quality of care, sometimes involving medical errors, and the consequent loss of confidence in the medical profession. Their quality of life is diminished. They have no plans, feel a certain emptiness and don’t see any positive aspect of their illness. Diagnosis announcements were difficult to live with and felt particularly abrupt. They are afraid of dependency and have difficulty accepting their illness. Figure 4 illustrates the themes related to psychological and existential changes of ideal-type 4 “psychological suffering”.

### Comparison between the ideal-types regarding PTGI and MQOL-R scores

When we compare the groups regarding the posttraumatic growth, we mainly observe a difference between the two groups characterized by better adaptation to change (“overcoming adversity” and “resilience”) and the two groups characterized by poorer adaptation (“Resigned acceptance” and “Psychological suffering”), with higher scores for the first two groups. This is particularly true for the total score, and the “new possibilities” and “appreciation for life” dimensions.

Regarding quality of life, the “Psychological suffering” group scored lower than the other three groups for the MQOL-R total score. The “overcoming adversity” group scored higher than the other three groups on the “feelings and thoughts” dimension while the “Psychological suffering” group scored lower on this dimension than the other three groups (see Table 2).

**Table 2:** Posttraumatic growth and quality of life scores for each ideal-types

	Overcoming Adversity (*n* = 6) Median (IQR), *n* (completed scales)		Resilience (*n* = 7) Median (IQR), *n* (completed scales)	Resigned acceptance (*n* = 3) Median (IQR), *n* (completed scales)	Psychological suffering (*n* = 7) Median (IQR), *n* (completed scales)
PTGI					
Personal Strength (0–20)		8 (3,7–13,5), 6	14 (3,5–16,5), 5	4,5 (4–5), 2	9 (2,5–11), 6
New Possibilities (0–25)		16,5 (5–17,5), 6	11 (2–16), 5	4,5 (4–5), 2	2 (0–8), 6
Improved Relationships (0–35)		19 (9,5–26,5), 5	21 (12,5–23,5), 5	13,5 (9–18), 2	22,5 (13,7–27,7), 6
Spiritual Growth (0–10)		2 (0–6), 6	5 (1,5–7), 5	2 (0–4), 2	0 (0-4.5), 6
Appreciation for Life (0–15)		11 (9,5–11,5), 5	7 (4,5–9), 5	4,5 (4–5), 2	5 (4–8), 5
Total score (0-105)		48,5 (28,2–66), 6	58 (27–69), 5	29 (23–35), 2	38,5 (18,2–53), 6
MQOL-R					
Physical (0–10)		56(4,5–6,8),5	5,3 (5,1–7,3), 5	5,8 (5–6,6), 2	4 (2,6 − 4,6), *n* = 7
Feelings and thoughts (0–10)		9,5 (4,5–10), 5	5,2 (4,5–6,1), 5	6,2 (5–7,5), 2	4,7 (3,2–7,2), 7
Existence (0–10)		7,6 ( -5,5–8,4), 4	6 (4,5–7), 5	6,6 (5–8,2), 2	5,7 (3,2–6,3), 7
Relationships (0–10)		6,6 (6–6), 5	6,6 (6,3–7,5), 5	6,3 (6–6,6), 2	6,3 (4,6–7), 7
Total score (0–10)		6,8 (5,5–8), 4	6 (5,4–6,5), 5	6,2 (5,2–7,2), 2	4,5 (3,9 − 6,5), 7

*IQR* Interquartile range, *PTGI*  Posttraumatic Growth Inventory, *MQOL-R* McGill Quality of Life-Revised questionnaire

## Discussion

The first aim of our study was to highlight the nature of the psychological changes experienced, including PTG, in the end-of-life context by drawing on the patients’ life storie. To collect patients’life story we used the Biographical Narrative Interpretive Method (BNIM) enabling to analyze three dimensions: a person’s full life history, the way they narrate it, and its social interpretation. It enables patients to recount their entire life trajectory; before, during, and after illness. By situating the individual within their life history and broader context, it provides a holistic understanding of their functioning beyond the sole context of illness, and highlights nuances that are often overlooked in standardized tools.

In order to analyze psychological changes experienced, including PTG, in the end-of-life context, we carried out two types of qualitative analysis: (i) an ideal-type analysis to categorize the patients according to distinct types of psychological changes; and (ii) a thematic content analysis to clarify the nature of these changes. The ideal-type analysis revealed four psychological profiles of patients. The first, “overcoming adversity” included several types of positive changes related to PTG, future projects, social support and satisfaction with the care context. The second, “resilience” showed fewer elements related to PTG but important coping strategies related to the management of the daily life activities. In the third profile, “Resigned acceptance”, patients showed acceptance toward the illness and serenity with the idea of dying despite the difficulties. The fourth profile, “psychological suffering” revealed significant physical and psychological suffering as well as despair and, for some patients, wishes for hastened death.

This diversity of profiles illustrates the various possible reactions to the progression of illness in an end-of-life context. All profiles showed some degree of acceptance of the illness. One of the most difficult aspects to come to terms with, specifically revealed by « Psychological suffering » and « Resigned acceptance», is loss of autonomy, which is known to impact a person’s dignity by affecting their identity, increasing their feeling of being a burden and/or of loss of control [25]. Seale and colleagues (1997) emphasized the importance of awareness of dying to maintain control. In our study, this is reflected in the recognition of life’s finitude and the desire to prepare for the end-of-life according to personal values. “Resigned acceptance” and " Psychological suffering " specifically revealed a wish to die and a loss of hope, while hope is a key driver of motivation and perseverance for people living with serious illness [26]. These findings further support the interpretation of the ideal-types as non-exclusive and overlapping patterns of psychological adaptation rather than fixed categories.

The four profiles go beyond a dichotomy consisting of PTG and resilience on one hand, and Psychological suffering and suffering on the other. The characteristics of the groups show unique distinctions but nevertheless share patterns of functioning. All groups include both factors of fragility and resources. The relative importance attributed to these factors was determinant for the classification of the patients. For instance, all profiles, except for “Psychological suffering”, revealed at least one change involving one of the five growth dimension described by Tedeschi and Calhoun [8]. Appreciation of life and simple pleasures, known to reduce distress and enhance well-being and quality of life [27], were the most common change reported by the patients. These results underline the complexity of individual adaptations in the face of illness, as well as the psychological and emotional fluctuations to which they are subject. They are consistent with the model developed by Keyes, who proposed that mental health does not work as a single continuum between health and illness. Mental health and illness are best apprehended as independent dimensions, which can co-exist at various levels of intensity within the same person [28]. This helps us to understand the painful process underlying posttraumatic growth. If the result is a positive experience for the patient, the internal journey is one of struggle and reflexive combat, which may well explain the presence of a certain distress in both the “Overcoming adversity” and “Resilience” profiles .

Consistent with findings reported in the literature, the results indicate that the dimensions of PTG are confirmed and enriched in the context of palliative care. Some dimensions take on a particular significance at the end of life, such as the “appreciation of simple pleasures” and “quality of interpersonal relationships”. Furthermore, new dimensions emerge beyond those documented in the literature, including the acceptance of finitude and preparation for death, attitudes toward loss of autonomy, functional adjustments related to illness, and the impact of healthcare professionals on patients’ psychological well-being.

Beyond the changes defining these profiles, differences emerge in how people construct life stories. The “Overcoming adversity” and “Resilience” profiles are characterized by chronological narratives that tend to begin with significant life events before the illness. As a result, individuals in these groups are more aware that they are not just patients at the end of life. They still embrace several roles (e.g., as part of their family or their professional lives), and have experienced successes and failures. This recognition can facilitate a deliberate self-analytical reflection, geared towards problem-solving to gain meaning from the initial traumatic or difficult experience. This way of reflecting has been identified as the most efficient process to facilitate adaptation and adjustment towards potential growth [29]. In contrast, patients in the “Resigned acceptance” and “Psychological suffering” profiles focus much more quickly on the illness or on certain moments of the illness (announcement of diagnosis, difficulty in coping with treatment), which can contribute to maintaining the “brooding” rumination process, a passive thoughtfulness about the causes and consequences of the initial trauma or suffering, which is likely to lead to less adaptation and more psychological suffering [30]. From the perspective of the posttraumatic growth model (Tedeschi & Calhoun), these differences may reflect variations in rumination processes. Patients in the “Resigned acceptance” and “Psychological suffering” profiles may experience difficulties in transforming intrusive rumination into more deliberate, meaning-oriented reflection, thereby limiting opportunities for cognitive restructuring and integration of the illness into their life narrative.

The second aim of our study was to compare these qualitative findings with quantitative assessments of quality of life and posttraumatic growth. In this regard, we analyzed quality of life and PTG of patients according to psychological changes profiles. The results of the quantitative analyses must be interpreted with caution, given the small size of the sample. An unexpected finding was that the “Resilience” profile showed a higher median PTGI total score than the “Overcoming adversity” profile. This result should be interpreted with caution given the small sample sizes within each subgroup (*n* = 3–7), which limit the robustness and stability of these descriptive comparisons. Rather than indicating a clear difference between these two profiles, this finding may reflect variability related to the small sample. From a conceptual perspective, this discrepancy may also be linked to differences between narrative expressions of change and self-reported perceptions captured by the PTGI. Participants in the “Resilience” profile, who tend to emphasize maintaining functioning and coping resources in the face of illness, may retrospectively endorse positive changes on standardized items, even in the absence of explicit transformation narratives. In contrast, the “Overcoming adversity” profile, characterized by more complex and sometimes ambivalent narrative processes, may reflect deeper or more nuanced transformations that are not fully captured by predefined questionnaire categories. This finding further supports the need to combine quantitative and narrative approaches when studying posttraumatic growth. However, our interpretation focuses primarily on the broader pattern distinguishing profiles with better psychological adaptation (“Overcoming adversity” and “Resilience”) from those with more limited adaptation (“Resigned acceptance” and “Psychological suffering”), rather than on differences between the two former groups. Overall, the PTGI total scores were globally moderate but revealed higher scores for the “Resilience” and “Overcoming adversity” profiles than to the others. This was particularly true for the dimensions “New opportunities” and “Appreciation of life” of the PTGI. These results tend to show some concordance between the posttraumatic growth assessed by means of a standardized questionnaire such as the PTGI, and by means of a qualitative life narrative approach. The latter, however, helps to identify more nuanced psychological processes that consider cognitive and emotional processes, as well as the internal struggles that may ultimately lead to the posttraumatic growth phenomenon.

While the PTGI scores tended to show a division between the “overcoming adversity” and “resilience” profiles on the one hand, and “Resigned acceptance” and “Psychological suffering” on the other, the MQOL-R total scores showed a gap between the first three profiles and the last profile, “Psychological suffering”, the latter reporting lower results. The dimension of quality of life for which the greatest differences between the groups are observed is the psychological dimension, with the “overcoming adversity” group standing out at the top and the “Psychological suffering” group at the bottom. As the groups were formed based on their psychological ability to adapt to the context, there is therefore a consistency between the qualitative data from the semi-structured interviews and the data from the questionnaire. The concept of quality of life encompasses broader dimensions than the psychological one (which is the case with the MQOL-R questionnaire). Nevertheless, these results tend to show that it is important not to underestimate the importance of psychological factors in assessing the global quality of life of patients at the end of life, as we were able to demonstrate in a previous study [31].

### Limitations and perspectives

Regarding the qualitative methodology, the Biographic-Narrative Interpretive Method represents a non-restrictive narrative method which gives participants more freedom to talk about their life. As discussed, the result is a great deal of variability in the way the life story is developed and constructed. Indeed, participants’ life stories varied in form, content, length, and structure, with heterogeneous experiences even within the same profile, complicating the comparative analytical process. The use of life narratives may have introduced a recall bias. Their current clinical condition and the palliative care context of the participants may also have retrospectively influenced the reconstruction of past experiences. In addition, the life narrative interview did not explicitly address posttraumatic growth, which may have contributed to limiting the influence of the initial interview on the responses given to the PTGI during the second interview (order effect), without cancelling it out entirely.

The methodological choice, based on a mixed-methods design and multiple analyses, also entailed certain difficulties. First the involvement of several researchers combining thematic and ideal-type analysis added further challenges in the analytical process. Second, Ideal-type analysis provides a structured typology that allows researchers to identify and categorize patients based on shared characteristics; however, it may oversimplify the complexity of individual experiences. By contrast, the thematic analysis, conducted using a codebook-based approach, allowed us to identify and characterize recurrent psychological and existential patterns across participants. While this approach does not aim to provide an in-depth interpretative account of individual meaning-making, it offers a more fine-grained description of similarities and differences within and across ideal-types. The richness of the data collected through the life story analysis revealed numerous additional themes that were not explored in this article, which specifically focuses on psychological changes in palliative care patients. To guide our thematic selection, we retained those most frequently represented within each profile. Accordingly, we established a threshold, admittedly subjective of ≥ 50% of participants per profile, for theme inclusion. Given the arbitrary nature of this criterion and its potential influence on the findings, this choice should be interpreted with caution. Other themes related to patients’ experiences of medical support, as well as their coping strategies and resources mobilized to face illness haven’t been studied in the present study but would be relevant avenues for future research.

Regarding the quantitative part, the small number of participants in each profile does not allow for statistical calculations that would enable conclusions to be drawn. However, we thought interesting to present the descriptive results to draw parallels with the profiles. These initial trends need to be corroborated with a larger sample.

Bridging ideal-type analysis, thematic analysis, and quantitative measures provided complementary insights, organizing diverse life narratives into coherent profiles, clarifying psychological and existential changes, and supporting qualitative distinctions with quantitative data. This mixed-methods approach compensated for the limitations of each method alone, offering a more comprehensive understanding of patients’ psychological adjustment at the end of life. Finally, participant’s recruitment was conducted exclusively within palliative care institutions in the French-speaking part of Switzerland, which may limit the representativeness of the sample and introduce selection bias.

Regarding the clinical context, we chose not to include variables such as disease stage, treatments received, and experiences of medical care due to the already large number of variables analyzed. However, these factors may have influenced the life narratives and could have either facilitated or hindered the process of posttraumatic growth, and deserve to be explored in future research.

### Clinical implications

In terms of clinical implications, the ideal-type analysis and the variability of the profiles identified highlight the value of taking patients’ life experiences into account, beyond the illness and end-of-life issues. Patients’ reactions to illness and their progress towards the last phase of life are strongly conditioned by their entire life trajectory. Healthcare professionals should be careful not to be biased by a single focus on the current hospital context, which may lead to believe that a serious and progressive illness could be the sole determinant of the patient’s adaptation and adjustment [32]. On several occasions, we observed that past life events represented major issues that could strongly condition the patient’s ability to cope and to adapt to the context of illness and end of life issues, influencing the quality of life. We encourage healthcare professionals to incorporate narrative interventions focusing on past life experiences into their clinical practice [30]. These interventions may not only serve as levers influencing one’s sense of meaning and coherence toward past experiences, but also foster adaptive processes in response to illness, which may, in some cases, give rise to posttraumatic growth. However, promoting PTG as an explicit clinical goal warrants caution. The process underlying PTG is often complex, effortful, and emotionally challenging, and not all patients may wish or be able to engage in it. Framing growth as an expected outcome may risk generating pressure or feelings of inadequacy in those who do not experience such changes. Rather, narrative approaches may be most appropriate when they support patients’ own meaning-making processes, including those who spontaneously engage in reflections that may lead to growth.

Furthermore, the process of disclosure, as used in narrative approaches, promotes cognitive restructuring. It thereby supports the development of adaptive strategies and problem-solving in response to new life circumstances, potentially contributing to post-traumatic growth (PTG). A literature review has shown that these approaches are feasible, including in the hospital context, as long as professionals demonstrate flexibility and creativity [32]. 

## Data Availability

The datasets analysed during the current study are available from the corresponding author on reasonable request.
